# Protective effect of aqueous extract from *Spirulina platensis *against cell death induced by free radicals

**DOI:** 10.1186/1472-6882-10-53

**Published:** 2010-09-21

**Authors:** Wan-Loy Chu, Yen-Wei Lim, Ammu Kutty Radhakrishnan, Phaik-Eem Lim

**Affiliations:** 1 International Medical University, No. 126 Jalan 19/155B, Bukit Jalil, 57000 Kuala Lumpur, Malaysia; 2 Institute of Biological Sciences & Institute of Ocean and Earth Sciences (IOES), University of Malaya, 50603 Kuala Lumpur, Malaysia

## Abstract

**Background:**

*Spirulina *is a commercial alga well known to contain various antioxidants, especially phycocyanin. Apart from being sold as a nutraceutical, *Spirulina *is incorporated as a functional ingredient in food products and beverages. Most of the previous reports on antioxidant activity of *Spirulina *were based on chemical rather than cell-based assays. The primary objective of this study was to assess the antioxidant activity of aqueous extract from *Spirulina *based on its protective effect against cell death induced by free radicals.

**Methods:**

The antioxidant activity of the cold water extract from food-grade *Spirulina platensis *was assessed using both chemical and cell-based assays. In the cell-based assay, mouse fibroblast cells (3T3) cells were incubated for 1 h in medium containing aqueous extract of *Spirulina *or vitamin C (positive control) at 25, 125 and 250 μg/mL before the addition of 50 μM 1,1-diphenyl-2-picrylhydrazyl (DPPH) or 3-ethylbenzothiazoline-6-sulfonic acid (ABTS). The cells were incubated for another 24 h before being assessed for cell death due to apoptosis using the Cell Death Detection ELISA Kit. Spectrophotometric assays based on DPPH and ABTS were also used to assess the antioxidant activity of the extract compared to vitamin C and vitamin E (positive controls).

**Results:**

*Spirulina *extract did not cause cytotoxic effect on 3T3 cells within the range of concentrations tested (0 - 250 μg/mL). The extract reduced significantly (p < 0.05) apoptotic cell death due to DPPH and ABTS by 4 to 5-fold although the activity was less than vitamin C. Based on the DPPH assay, the radical scavenging activity of the extract was higher than phycocyanin and was at least 50% of vitamin C and vitamin E. Based on the ABTS assay, the antioxidant activity of the extract at 50 μmug/mL was as good as vitamin C and vitamin E.

**Conclusions:**

The results showed that aqueous extract of *Spirulina *has a protective effect against apoptotic cell death due to free radicals. The potential application of incorporating *Spirulina *into food products and beverages to enhance their antioxidant capacity is worth exploring.

## Background

Free radicals and reactive oxygen species (ROS) such as hydroxyl radical (HO.), superoxide radical (O_2_.^-^), peroxyl radical (ROO.), nitric oxide radical (NO.) and hydrogen peroxide (H_2_O_2_) are highly reactive molecules produced from aerobic metabolism. Such oxidants can damage the cellular membrane or intracellular molecules (especially DNA) if not efficiently removed by the antioxidant defense mechanisms of the cell [1]. Free radicals and ROS are associated with important pathological processes including inflammation, neurodegenerative diseases, artherosclerosis and cancer [2]. There has been much interest in exploiting antioxidants from natural sources as there is concern over the toxic effects of synthetic antioxidants.

*Spirulina *is a blue-green alga (cyanobacterium) that has been consumed as food since ancient times by the Mexicans and natives in the Lake Chad area. The alga is presently marketed as a food supplement (nutraceutical) due to its high contents of proteins, γ-linolenic acid, vitamins and minerals. There have been many reports on the therapeutic implications of *Spirulina*, including for health problems like diabetes, arthritis, anaemia, cardiovascular diseases and cancer [3-6]. *Spirulina *is also incorporated into various food products to enhance their nutritional qualities and the preparations will be useful in therapeutic management of chronic disorders such as diabetes, hypertension and heart disease [7].

*Spirulina *is well-known to have antioxidant properties, which are attributed to molecules such as phycocyanin, β-carotene, tocopherol, γ-linolenic acid and phenolic compounds [8]. For instance, selenium-containing phycocyanin from *Spirulina *has been shown to have strong superoxide and hydrogen peroxide radical-scavenging activities [9]. *Spirulina *extract is known to have higher antioxidant activity than another commercial alga, *Chlorella *due to its higher content of phenolic compounds [10]. Ingestion of *Spirulina *showed preventive effect against the skeletal damage under exercise-induced oxidative stress [11]. In addition, *Spirulina *has been shown to have protective effects against oxidative-stress induced by lead acetate in the liver and kidney of rats [12]. Feeding of *Spirulina platensis *also reduces hepatoxicity induced by cadmium in rats and the effect is suggested to be mediated through its antioxidant properties [13]. *Spirulina *is also known to have protective effects against nephrotoxicity due to oxidative damage induced by gentamicin [14].

There have been very few studies on the antioxidant activity of *Spirulina *using cell-based assays. One of such studies used neutrophils to assess the antioxidant and anti-inflammatory activities of *Spirulina platensis *preparations [15]. In comparison, cell-based assays have been used to assess the protective effect of other antioxidants. For instance, cell-based assays using mouse fibroblast cells (3T3) and mouse mammary gland tumour cells (4T1) were used to assess the antioxidant activity of the extracts from *Nephelium lappaceum *[16]. In another study, Seo et al. (2009) [17] assessed the protective effect of lycopene against oxidative stress-induced cell death of pancreatic acinar cells. Other antioxidant studies were mainly based on spectrophotometric methods which measure scavenging activities of radicals such as 1,1-diphenyl-2-picrylhydrazyl (DPPH) and 3-ethylbenzothiazoline-6-sulfonic acid (ABTS) [10,18]. The objective of the present study was to investigate the protective effect of aqueous extract from *Spriulina *against cell death induced by the free radicals ABTS and DPPH using mouse fibroblast cells (3T3) as a model. In comparison, chemical assays based on DPPH and ABTS were also conducted.

## Methods

### Samples, cell lines and chemicals

The food-grade *Spirulina platensis *powder used in this study was provided by Siam Algae Company (Thailand). The mouse embryo fibroblast cells 3T3 (NIH) were purchased from the American Tissue Culture Collection (ATCC). The culture medium used was RPMI 1640 with L-glutamine, fetal serum albumin, penicillin and streptomycin (GIBCO, Invitrogen Corp., New Zealand). The chemicals 1-diphenyl-2-picrylhydrazyl (DPPH) and 3-ethylbenzothiazoline-6-sulfonic acid (ABTS) were obtained from Sigma Chemicals (USA) and Calbiochem (USA) respectively. The antioxidants vitamin C and vitamin E used as positive controls were obtained from Sigma Chemicals (USA). For comparative purposes, phycocyanin (Sigma Chemicals) was included in the study. Dimethylsulfoxide (DMSO) of tissue culture grade (Sigma Chemicals) was used in this study.

### Preparation of extracts

The *Spirulina *powder (20 g) was soaked in 1 L of ultrapure water and shaken continuously for 24 h at room temperature. The mixture was then centrifuged at 5,000 rpm for 10 min (4°C) and the supernatant was filtered (Whatman No. 1) to remove the cell debris. The sample was then freeze-dried and the dried extract was stored at 4°C before use for the experiments.

### Cytotoxicity testing of *Spirulina *extract

Cytotoxicity testing was conducted to determine the range of concentrations of extract to be used for the cell-based antioxidant assay. The 3T3 cells (6 × 10^4 ^cells/mL) were incubated (5% CO_2_, 37°C) in 96-well plates containing 0 to 250 μg/L *Spirulina *extract for 72 h. Cell viability was determined at 72 h using the XTT Cell Proliferation Kit II as described by the manufacturer (Roche Applied Science, Germany). The principle of this test kit is based on the metabolism of yellow tetrazolium salt to a water soluble orange formazon dye by viable cells. By measuring the absorbance of the samples (450 nm), the percentage of viable cells could be determined.

### Determination of the viability of cells treated with DPPH and ABTS

The cell suspension (50 μL) was mixed with 50 μL ABTS or DPPH in the multi-well plate. The final ABTS concentrations ranged from 0 to 200 μM in 0.3% ethanol while the final DPPH concentrations ranged from 0 to 50 μM in 0.3% DMSO. The wells containing cells without ABTS or DPPH served as negative control. The cultures were incubated (5% CO_2_, 37°C) for 24 h before the determination of cell viability using the XTT Cell Proliferation Kit II.

### Apoptotic assay

Confluent 3T3 cells were harvested and plated in 96-well plate, with each well containing 50 μL cell suspension (1 × 10^5 ^cells/mL). The *Spirulina *extract (50 μL) was added to each well at final concentrations of 25, 125 and 250 μg/mL. The negative control consisted of cells without the extract. The plates were then incubated for 1 h in a humidified 5% CO_2 _incubator at 37°C before the determination of apoptotic cell death using the Cell Death Detection ELISA kit.

After incubation, the cell suspension was centrifuged (1200 rpm, 10 min) and the supernatant removed. The cell pellet was treated with lysis buffer for 1 h before being centrifuged again (1200 rpm, 10 min). The cell lysate was used for apoptosis assay with the ELISA kit, which determined specifically mono- and oligonucleosomes in the cytoplasmic fraction. The enrichment of mono- and oligonucleosomes in the cytoplasm of apoptotic cells was determined based on the absorbance at 405 nm using the following formula: Enrichment factor = absorbance of the sample (treated)/absorbance of the control (non-treated).

The above procedures were repeated to assess the protective effect of *Spirulina *extract and vitamin C against apoptotic cell death induced by ABTS and DPPH. A volume of 50 μL of ABTS or DPPH was added to each well at final concentrations of 50 and 20 μM respectively after incubation for 1 h in medium containing vitamin C or *Spirulina *extract at 25, 125 and 250 μg/mL. The plates were incubated for another 24 h before apoptotic cell death was assessed.

### Determination of antioxidant activity

The antioxidant activity of the algal extracts was determined based on DPPH radical-scavenging activity [19] and ABTS radical decolouration assay [20]. In the DPPH assay, 50 μL *Spirulina *extract was mixed with 50 μL of 500 μM DPPH in ethanol and kept in the dark for 40 min. The absorbance of the mixture was measured at 492 nm. Vitamin C and vitamin E were used as positive controls. The radical scavenging activity was determined based on percentage inhibition of absorbance, which was calculated using the following formula:

$$\text{Percentage inhibition of absorbance}=[{\text{OD}}_{492}(\text{DPPH}+\text{ethanol})-{\text{OD}}_{492}\text{ sample}]/[{\text{OD}}_{492}(\text{DPPH}+\text{ethanol})]\times 100\%$$

In the ABTS assay, 715 μL ABTS solution was mixed with 285 μL potassium persulfate (2.45 mM) in a microcentrifgue tube to a concentration of 5 mM. The reaction mixture was left in the dark for 12 to 16 h to generate ABTS free radicals. After this activation step, the mixture was diluted with 0.01 M phosphate buffered saline (PBS) at pH 7.4 to give an absorbance value of 0.70 ± 0.02 at 734 nm. This was followed by adding 10 μL of test compound (*Spirulina *extract, vitamin C or vitamin E) at 25 and 50 μg mL^-1^. Absorbance was measured at 734 nm at 1 min interval for 15 min. The percentage reduction of absorbance at 15 min compared to the initial value was determined.

### Statistical analysis

All the experiments were run in triplicate. Data from the cell death assays were analysed by paired-sample T-test with p value set at 0.05 (SPSS software version 11.5). Data from the assay on ABTS-scavenging activity were analysed by one-way anlysis of variance (ANOVA) followed by Duncan's Multiple Range test. A value of p < 0.05 is considered significant.

## Results

### Effect of *Spirulina *extract on cell viability and apoptotic cell death

**Figure 1:**
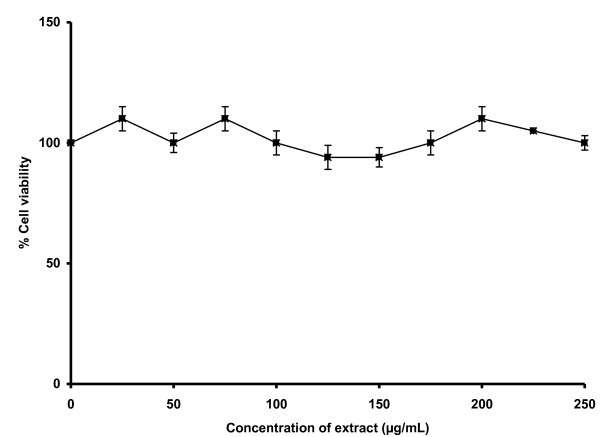
**Effect of *Spirulina *extract on the viability of 3T3 cells**. The cells were incubated with the extract for 72 h before being assessed for their viability. Data presented as mean ± standard deviations (n = 3).

**Figure 2:**
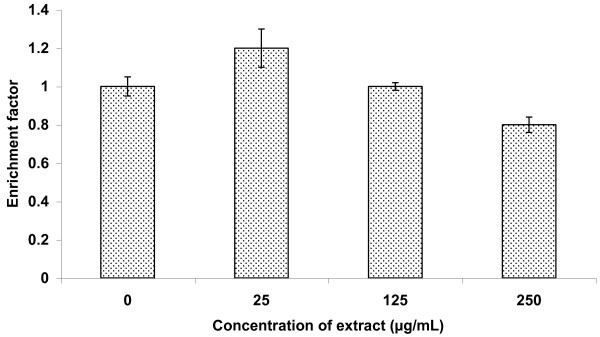
**Effect of *Spirulina *extract on apoptotic cell death of 3T3 cells**. The cells were incubated for 1 h before being assessed for apoptosis. Data presented as mean ± standard deviation (n = 3). The enrichment factors of the cells treated with extract were not significantly different (p > 0.05) from the control.

The viability of 3T3 cells was not significantly reduced by the *Spirulina *extract up to 250 μg/mL (Figure 1). Therefore, three concentrations of extract, namely 25, 125 and 250 μg/mL within this range were used for further testing in the cell-based assay. The extract did not cause any significant (p > 0.05) effect on cell death due to apoptosis compared to the untreated cells (Figure 2).

### Effect of DPPH and ABTS on cell proliferation

**Figure 3:**
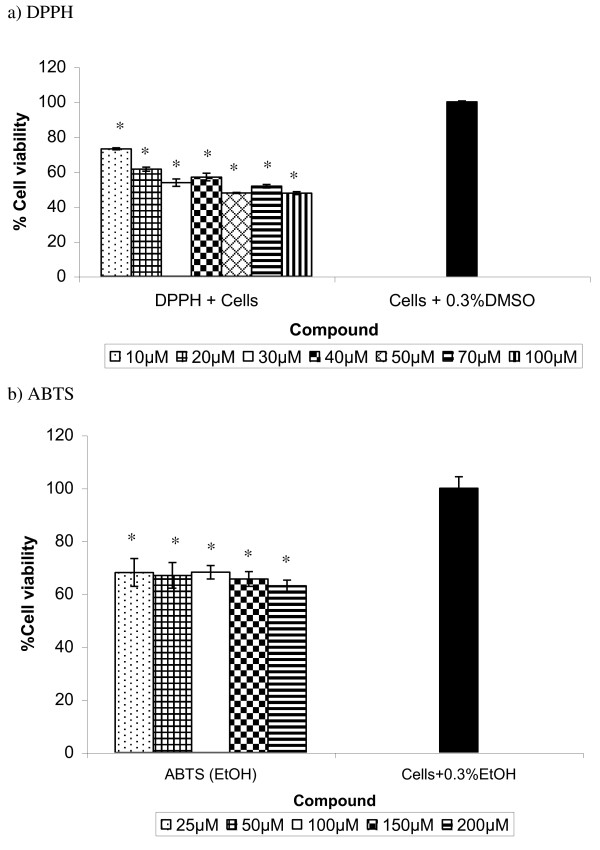
**Effect of a) DPPH and b) ABTS on the viability of 3T3 cells**. The cells were incubated with DPPH or ABTS for 24 h before being assessed for their viability, compared to those treated with dimethyl sulfoxide (DMSO) or ethanol (EtOH) alone (negative control). Data presented as mean ± standard deviation (n = 3). *Denotes significant difference (p < 0.05) from the control (cells + 0.3% DMSO/EtOH).

The DPPH at 10 to 100 μm killed 30 - 50% of the cells after 24 h incubation (Figure 3a). In comparison, the cell viability was reduced by 35 - 40% after being treated with ABTS (25 - 200 μm) for 24 h (Figure 3b).

### Protective effect of *Spirulina *extract against apoptotic cell death due to DPPH and ABTS

**Figure 4:**
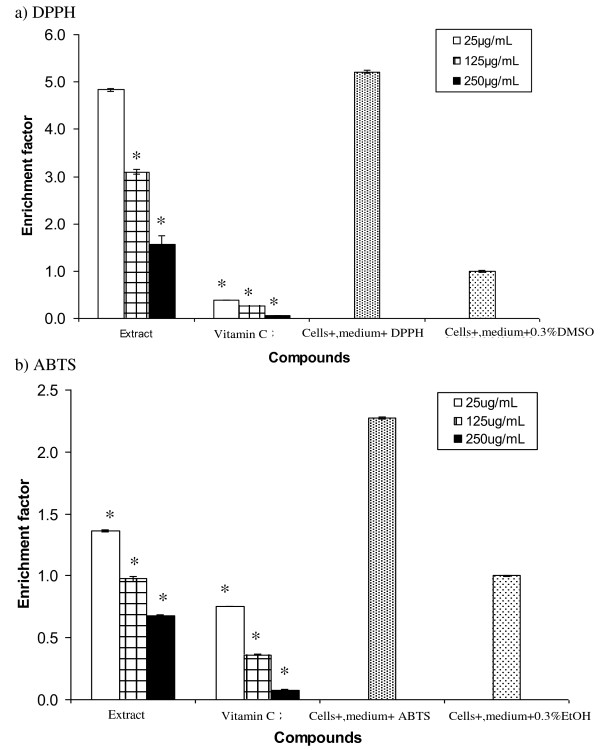
**Protective effect of *Spirulina *extract against apoptotic cell death induced by a) DPPH and b) ABTS**. The 3T3 cells were incubated in medium containing *Spirulina *extract or vitamin C for 1 h before being added with 20 μM DPPH or 50 μM ABTS and incubated for another 24 h before the determination of apoptotic cell death. The positive control contained cells incubated with vitamin C while the negative control contained medium alone. Data presented as mean ± standard deviation (n = 3). *Denotes significant difference (p < 0.05) from "cells + medium + DPPH/ABTS".

*Spirulina *extract at 125 and 250 μg/mL reduced significantly (p < 0.05) apoptotic cell death induced by DPPH (Figure 4a). At 250 μg/mL, the extract reduced the DPPH-induced apoptotic cell death by almost five-fold. However, the activity was still less than vitamin C. In comparison, the extract was effective in reducing apoptotic cell death due to ABTS even at the lowest concentration (25 μg/mL) (Figure 4b). At 250 μg/mL, the extract reduced apoptotic cell death due to ABTS by almost 40% although the activity was less than vitamin C.

### Antioxidant activity based on DPPH and ABTS assays

**Figure 5:**
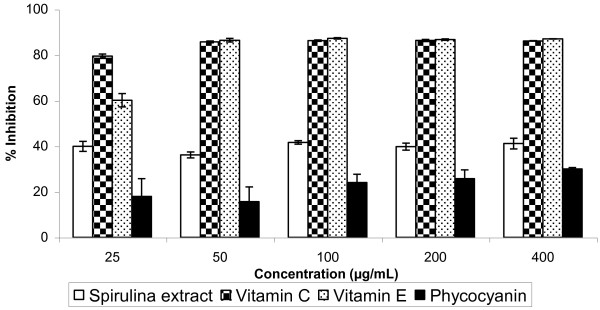
**DPPH-scavenging activity of *Spirulina *extract compared with vitamin C, vitamin E and phycocyanin**. Data presented as mean ± standard deviation (n = 3).

**Figure 6:**
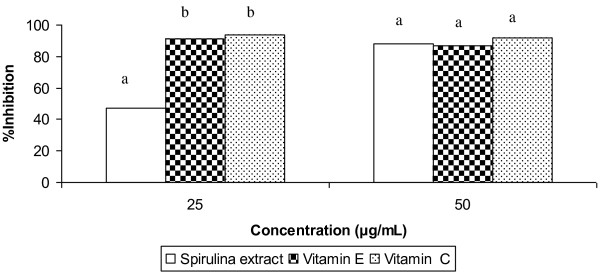
**ABTS-scavenging activity of *Spirulina *extract compared with vitamin C and vitamin E at 25 and 50 μg/mL**. Data presented as mean values from three replicates; standard deviations < 1% of means. Different alphabets denote significant difference (p < 0.05) at each concentration.

The DPPH assay showed that the antioxidant activity of *Spirulina *extract was approximately 50% of vitamin C and vitamin E (Figure 5). In comparison, the antioxidant activity of the extract was higher than phycocyanin, which is the major pigment of *Spirulina*. Based on the ABTS assay, the antioxidant activity of the extract at 25 μg/mL was about 50% of vitamin C and vitamin E (Figure 6). At 50 μg/mL, the antioxidant activity of the extract was similar to vitamin C and vitamin E.

## Discussion

Various assays have been used to assess the antioxidant activity of *Spirulina *extracts and compounds. Most of the *in vitro *assays are based on the measurement of radical scavenging activity [9,10,18] while rat models [21] and human subjects [11] are often used in *in vivo *studies. The present study used a combination of both chemical and cell-based assays to assess the antioxidant activity of *Spirulin*a extract.

Aqueous extract rather than pure compounds from *Spirulina *was used in this study as the whole biomass is consumed or added as a functional ingredient in food products and beverages. It will be beneficial if the inclusion of *Spirulina *does increase the antioxidant property of such products. This will be an additional health benefit as *Spirulina *contains a wide range of other nutrients such as γ-linolenic acid, β-carotene and proteins.

The *Spirulina *extract alone did not impose significant effect on apoptotic death in cells that were not first treated with free radicals. This is beneficial as it suggests that *Spirulina *extract does not cause apoptotic death in normal cells which have not been exposed to increased oxidative stress. The extract also did not reduce apoptotic cell death if there was no prior treatment with free radicals. This trend differed from that reported by Palamisamy et al. (2008) [16] who tested the extracts of the rambutan *Nephelium lappaceum *on the same cell line, which reduced apoptotic cell death significantly even without prior induction by free radicals.

The observation also differed from Wu et al. (2005) [10] who showed that aqueous extract of *Spirulina *induced apoptosis in hepatic stellate cells (HSC). However, the regulation of the cell cycle of HSC by the extract may not be due its antioxidant activity. Instead, it could be due their effect on the activity of tyrosine kinase receptor and the expression of the cell cycle protein cyclin D1. *Spirulina *extract enriched with selenium has been shown to inhibit the growth of breast cancer cells through induction of apoptosis [22]. Thus, the effect of *Spirulina *extract on apoptosis varies with the type of cells used for the test.

The results showed that the extract has protective effect against cell death due to apoptosis induced by DPPH and ABTS. The extract might exert its effect by scavenging the free radicals and this might reduce the activation of the apoptotic pathway [23]. The extract might also exert its effect by reducing the oxidative damage due to free radicals. Recently, it has been shown that phycocyanin from *Spirulina *reduces apoptotic cell death of pancreatic beta cells by preventing the overproduction of reactive oxygen species and enhancing the activities of superoxide dismutase and glutathione peroxidase [24].

Apoptotic cell death occurs due to the activation of various pathways that involve caspases, ceramide, altered gene expression, mitochondrial dysfunction and consumption of ATP that result in DNA fragmentation [25]. Many of the pathways are associated with the production of oxidants that contribute to the magnitude of cell death. Antioxidants may protect against apoptotic cell death by preventing the loss of DNA repair proteins such as Ku70 and Ku80, as shown by the action of lycopene in pancreatic acinar cells [17]. Increase in apoptotic cell death often occurs in pathological conditions such as inflammation or infection and diseases such as Alzeimer's disease, Parkinson's disease and diabetes mellitus [26]. Thus, the antioxidant effect of *Spirulina *extract could be useful in ameliorating such pathological conditions. Dietary antioxidants from sources such as green tea and cat's claw have been shown to limit epithelial cell death in the intestine due to oxidative stress [27]. In the present study, mouse fibroblast cells were used a model for epithelial cells. Protective effect against radical-induced apoptosis in epithelial cells may have the potential in reducing pathological effects associated with cell death resulting from inflammatory reactions, which are linked to oxidative stress.

Phycocyanin is the major water-soluble antioxidant constituent in *Spirulina*, with its activity about 20 times more efficient than vitamin C [8]. The covalently-linked tetrapyrole chromatophore phycocyanobilin is suggested to be involved in the scavenging activity of phycocyanin [28]. Phycocyanin has been shown to protect normal human erythrocytes and plasma against oxidative damage in *in vitro *studies [29]. In addition, phycocyanin protects pancreatic beta cells against apoptotic cell death by attenuating oxidative stress [24]. The present study showed that the antioxidant activity of phycocyanin was less than *Spirulina *extract and vitamin C. The extract might contain other constituents (e.g. phenolic compounds) which gave a higher combined antioxidant activity than phycocyanin alone. The synergistic action of a wide spectrum of antioxidants may be more effective than the activity of a single antioxidant.

As the radical system used for antioxidant evaluation may give different results, two or more radical systems are required to assess the radical-scavenging activity of an antioxidant [30]. In the present study, DPPH and ABTS assays were used to assess the antioxidant activity of the extracts. The radical scavenging activity of the extract was higher in the ABTS assay than the DPPH assay at 50 μg/mL. Similar observation was reported for the methanol extracts from the higher plant *Calpurnia aurea *[31]. In contrast, some antioxidant compounds which show ABTS scavenging activity may not have DPPH scavenging activity, as found in phenolic compounds from sage [32]. The difference in antioxidant activity based on the two assays could be due to the different mechanisms in scavenging ABTS and DPPH radicals. In the DPPH assay, the antioxidant effect was likely to be due to the hydrogen donating ability of the extract [33]. The ABTS assay is a measure of the activity of the antioxidant in scavenging proton radicals through donation of electrons [34]. Furthermore, factors such as stereoselectivity of the radicals and the solubility of the extract in different testing systems may also affect its capacity to quench different radicals [29]

## Conclusion

This study showed that the aqueous extract of *Spirulina *could reduce significantly apoptotic cell death induced by the free radicals DPPH and ABTS. The antioxidant activity of the extract was much higher than phycocyanin based on the DPPH assay, suggesting that a mixture of compounds is more active than a single pure compound. Further studies to compare the activity of single and mixed antioxidant compounds from *Spirulina *are worthwhile. The potential of incorporating *Spirulina *extract as a functional ingredient in food products and beverages to enhance their antioxidant capacity is worth exploring.

## Competing interests

The authors declare that they have no competing interests.

## Authors' contributions

CWL prepared the manuscript and coordinated the study. LYW carried out the experiments. Both AR and LPE were involved in designing the experiments. All authors read and approved the final manuscript.

## Pre-publication history

The pre-publication history for this paper can be accessed here:

http://www.biomedcentral.com/1472-6882/10/53/prepub
